# Characterization of the Blood and Cerebrospinal Fluid Microbiome in Children with Bacterial Meningitis and Its Potential Correlation with Inflammation

**DOI:** 10.1128/mSystems.00049-21

**Published:** 2021-06-08

**Authors:** Huiping Liao, Yuchao Zhang, Wei Guo, Xi Wang, Hailong Wang, Haocheng Ye, Kai Wu, Yu-Hang Zhang, Lingyun Guo, Yufei Zhu, Yongli Guo, Landian Hu, Gang Liu, Xiangyin Kong

**Affiliations:** a Key Laboratory of Molecular Genetics, Shanghai Institute of Nutrition and Health, University of Chinese Academy of Sciences, Chinese Academy of Sciences (CAS), Shanghai, China; b Key Laboratory of Major Diseases in Children, Ministry of Education, Department of Infectious Diseases, Beijing Children’s Hospital, Capital Medical University, National Center for Children’s Health, Beijing, China; c Research Unit of Critical Infection in Children, Chinese Academy of Medical Sciences, Beijing, China; University of California, San Francisco; Javelin Biotech, Inc.

**Keywords:** bacterial meningitis, children, metagenomics, microbiome

## Abstract

Bacterial meningitis shows a higher incidence in children than adults, but signs may be scarce. Although some pathogenic microorganisms of meningitis from cerebrospinal fluid (CSF) have been reported, the signature of the representative microbiota in CSF and blood samples from patients remains incompletely revealed. To extend the understanding of the microbiome in patients, we recruited 32 children with bacterial meningitis, 30 undiagnosed infectious children, and 10 matched healthy individuals, which was followed by untargeted metagenomic next-generation sequencing (mNGS) and bioinformatic analysis. Our results showed that children with bacterial meningitis exhibited different microbiome signatures in their CSF and blood compared with undiagnosed and healthy children, and patients could be divided into varied subsets according to these signatures, including Escherichia coli, Klebsiella pneumoniae, Thermothelomyces thermophila, Lactobacillus acidophilus, and Staphylococcus haemolyticus. To further explore their potential role in patients’ conditions, we examined their correlation with clinical parameters. Importantly, microbiome signatures with compositional changes were correlated with the C-reactive protein (CRP) level in blood and granulocyte percentage in CSF. Moreover, the blood in subsets of patients with a predominance of Klebsiella pneumoniae could replace CSF as the main specimen for clinical monitoring.

**IMPORTANCE** This study revealed the microbial compositions in children with bacterial meningitis who were treated with antibiotics and made a comprehensive comparison between blood and CSF specimens for the risk and prognosis assessment. We found that microbiome signatures could distinguish patient subsets in the children and were correlated with the CRP level in blood and granulocyte percentage in CSF. The compositional changes in representative microbiota constituents could provide guidance for clinical monitoring and antibiotic intervention.

## INTRODUCTION

Bacterial meningitis (BM) is a damaging infectious disease of the central nervous system in children, especially infants (1), and its incidence in developing countries, such as Africa, is much higher than that in developed countries (2). With the development of vaccinations for meningococcus, Haemophilus influenzae, and Streptococcus pneumoniae, the mortality of this disease has been significantly reduced, but it is still between 5% and 15% (3, 4).

Early and accurate diagnosis of BM is vital for implementing an effective therapeutic schedule, such as antibiotic treatment. However, it is often impossible due to the limitations of traditional approaches in terms of sensitivity and the time requirements of culture and bacterial cultivation. The current standard for diagnosing infections depends on differential patient history, clinical manifestations, and imaging detection, followed by laboratory testing. This conventional method is particularly challenging for meningitis due to the lack of diagnostic tests for uncommon pathogenic microorganisms and the limited availability and volume of cerebrospinal fluid (CSF) samples because of the invasiveness of the procedures, such as lumbar puncture. Hence, the cause of meningitis remains unexplained in a large proportion of patients (5).

Metagenomic next-generation sequencing (mNGS) has a comprehensive performance in verifying nearly all probable microorganisms, including bacteria, viruses, fungi, and parasites, in a single test (6–9). However, most published studies mainly described the suitability of mNGS for the diagnosis of pathogenic bacteria in individual cases or provide diagnostic reports (10–13). There is no study focusing on the microbial signatures of patients with BM with drug intervention. A better understanding of the compositional changes in microbiomes will largely contribute to revealing the role of pathogens in BM.

We performed a prospective study involving hospitalized children diagnosed with BM. Recently, the sensitivity and specificity of mNGS of CSF have been proven in the identification of pathogens in the central nervous systems of infected patients (7, 14). Our study was designed to evaluate the specific signatures of the microbiota detected by mNGS in the CSF and blood from children with meningitis confirmed by conventional tests and who underwent antibiotic treatment. Our analysis demonstrated that microbiome signatures are different in BM patients, undiagnosed children, and healthy individuals. These microbiome signatures included Escherichia coli, Klebsiella pneumoniae, Thermothelomyces thermophila, Lactobacillus acidophilus, and Staphylococcus haemolyticus, and their composition patterns can divide patients into different subgroups. Their correlation with clinical parameters was assessed to further explore the potential role of representative microbiota constituents in patients’ conditions. The results showed that microbiome composition changes were associated with the C-reactive protein (CRP) levels in blood and granulocyte percentages in CSF. Furthermore, the subsets of patients with a predominance of Klebsiella pneumoniae could instead use blood for clinical monitoring. These results provide a significant reference for clinical monitoring and antibiotic treatment.

## RESULTS

### Study overview and analysis workflow.

**FIG 1:**
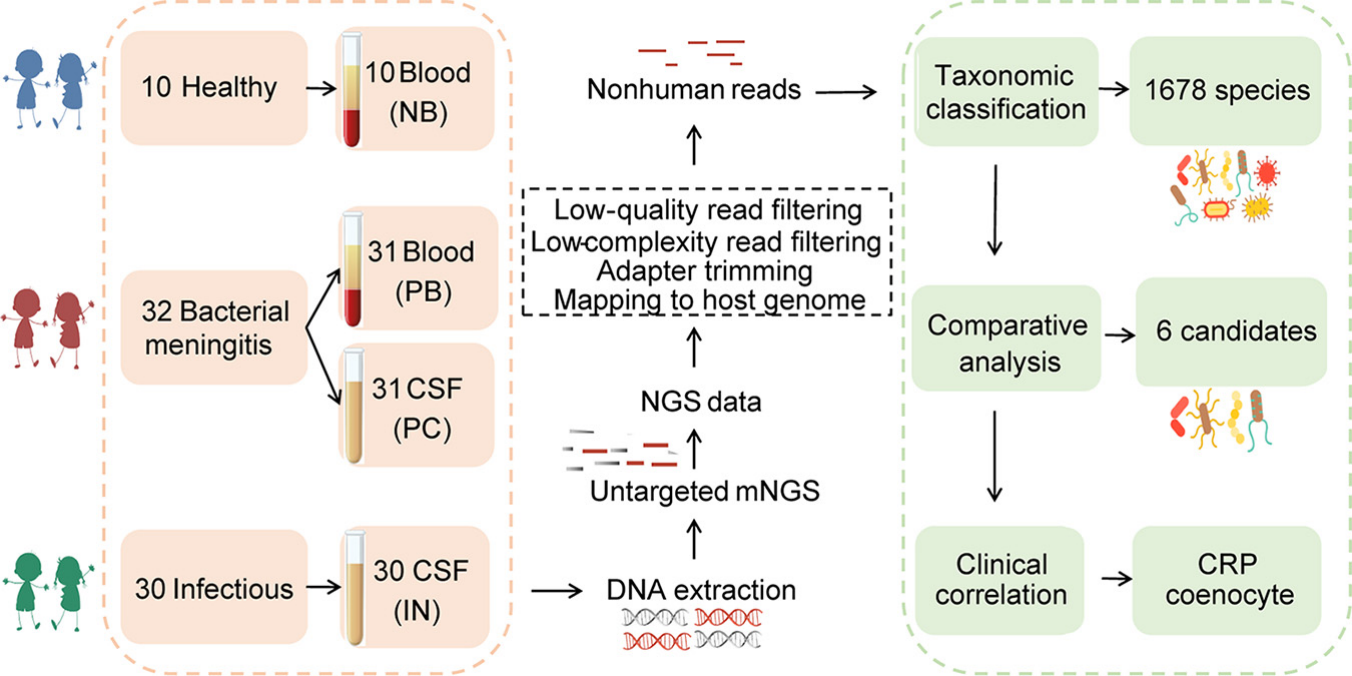
Study overview and analysis workflow. Patients with bacterial meningitidis (BM) and healthy children were enrolled, and blood and CSF samples were collected and subjected to DNA sequencing analysis by untargeted mNGS methods for pathogen identification.

We enrolled 32 children with BM, 30 infected but undiagnosed individuals, and 10 healthy controls for this microbiome study. Notably, all children with BM received antibiotic treatment and were discharged upon improvement. A total of 102 DNA samples were extracted from specimens: 10 from healthy blood (NB), 31 from patient blood (PB), 31 from patient CSF (PC), and 30 from CSF from undiagnosed patients (IN) (Fig. 1, red boxes). Considering the impermeability of the blood-brain barrier (BBB) and the blood-CSF barrier (BCSFB) to microorganisms (15), we mainly focused on the comparison of recruiter groups based on blood and cerebrospinal fluid. Follow-up studies mainly consisted of taxonomic classification, comparative analysis, and clinical association analysis (Fig. 1, green boxes).

### Children with bacterial meningitis harbor distinct microbiome communities in their blood and CSF compared with those in healthy controls and undiagnosed individuals.

**FIG 2:**
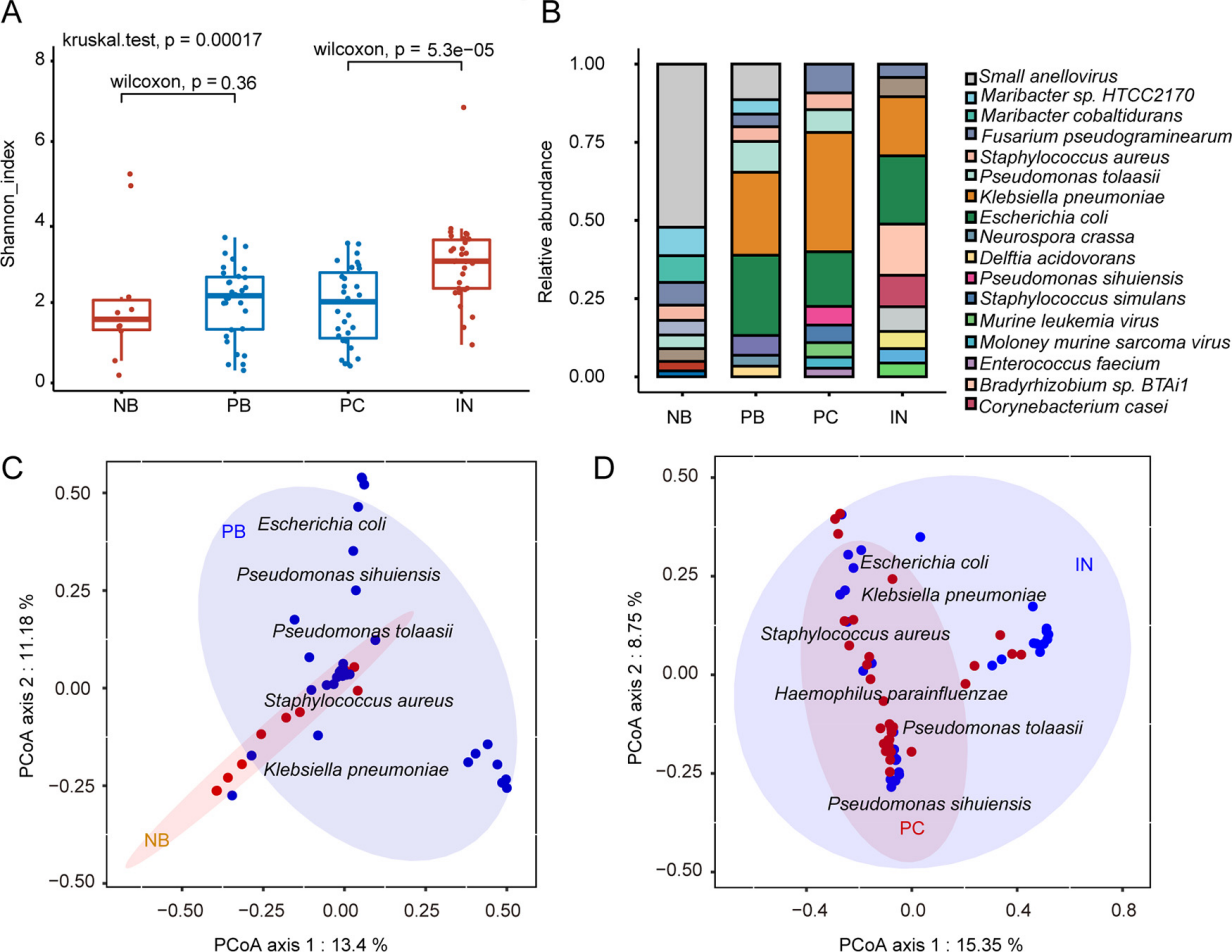
Children with bacterial meningitis harbor a distinct microbiome. (A) Boxplots of microbial α-diversity measures (Shannon index) for blood from healthy controls (NB) and blood (PB), and CSF from BM patient (PC) and undiagnosed cases (IN). A two-sided Wilcoxon rank sum test and Kruskal-Wallis test were used to compute *P* values (Shannon and Simpson indexes; see also Fig. S1 in the supplemental material). (B) Species-level distribution of the microbiota in the four groups in CSF and plasma. The numbers of replicated samples in this figure are as follows: *n* = 10 for NB, *n* = 31 for PB, *n* = 31 for PC, and *n* = 30 for IN. (C) PCoA plot of the microbiota using the Bray-Curtis distance metric of β-diversity in the blood of meningitis patients and healthy controls. (D) The ellipses indicate the 95% confidence intervals. The text denotes the locations of the top contributing species, and weighted average scores were computed by the wascores function.

To explore the microbiome composition in BM, we measured microbial diversity and found that within-sample diversity (α-diversity) displayed a significant difference (*P* = 5.3e−05 and *P* = 0.0078 for Shannon and Simpson indexes, respectively) between patient CSF (PC) and undiagnosed individuals’ CSF (IN) (Fig. 2A; see also Fig. S1 in the supplemental material). The microbiota of undiagnosed patients had higher α-diversity than that of BM patients in CSF, while there was no significant difference between healthy controls and BM patients in blood (Fig. 2A). This result showed that antibiotic treatment in BM patients caused the ablation of bacteria. The differences in the relative abundances of predominant microbiota constituents between patients (PB, PC, and IN) and controls (NB) were significant and detectable at the species level (Fig. 2B). In all patients, higher relative abundances of Klebsiella pneumoniae, Escherichia coli, Pseudomonas tolaasii, and Staphylococcus aureus were observed in patients than in healthy controls, whereas the relative abundance of small anelloviruses was higher in healthy individuals. On the basis of these results, we then tested the differentially abundant pathogens in the blood and CSF groups. As expected, we found that the composition of the microbiomes varied in BM samples versus healthy samples. Unconstrained principal coordinate analysis (PCoA) of Bray-Curtis distance revealed that the microbiota of healthy controls (NB) and patients (PB) in blood formed two distinct clusters (Fig. 2C). The largest principal contributors of variation in the blood microbiota were Escherichia coli, Klebsiella pneumoniae, Pseudomonas tolaasii, Pseudomonas sihuiensis, Staphylococcus aureus, and small anellovirus. However, the overall species composition was different between BM children (PC) and undiagnosed patients (IN) (Fig. 2D). Our findings indicated that both the components and abundances of the blood microbiota were different in BM patients and healthy individuals, and the CSF microbiota exhibited differences in diversity and components between BM patients and undiagnosed patients.

10.1128/mSystems.00049-21.1 FIG S1 Diversity comparison among different groups. (A) Comparison of Shannon index of each group, comparison between two groups using Student’s *t* test (t test), comparison of multiple groups using Kruskal-Wallis test. (B) Comparison of Shannon index of each group, comparison of two groups using Wilcoxon test, comparison of multiple groups using Kruskal-Wallis test. (C) Comparison of Simpson index of each group, comparison between two groups using Student’s *t* test (t test), comparison of multiple groups using Kruskal-Wallis test. (D) Comparison of Simpson index of each group, comparison between two groups using Wilcoxon test, comparison of multiple groups using Kruskal-Wallis test. Download FIG S1, TIF file, 1.4 MB. Copyright © 2021 Liao et al. 2021 Liao et al. https://creativecommons.org/licenses/by/4.0/ This content is distributed under the terms of the Creative Commons Attribution 4.0 International license.

### Representative microbiota constituents related to meningitis can be regarded as signatures in patients.

Considering the different microbial compositions in blood and CSF, we wondered whether microbiota members can be used as biomarkers to differentiate NB and PB groups as well as PC and IN. To this end, we established a model using a random-forest machine learning method (16) to correlate patients and controls with microbiota data in the blood and CSF at the species level. We carried out 10-fold cross-validation with five repeats to evaluate the importance of indicator bacterial species. Several pathogenic bacteria (Table 1) were identified with mean decrease accuracy (MDA) value of 0.43 in blood and 0.002 in CSF. The higher MDA value of bacterial species enriched in IN was consistent with the observation that IN samples showed higher microbial diversity. Recent studies have proposed that edgeR has excellent performance in the identification of differentially abundant genes in metagenomes (17).

**TABLE 1:** Candidate species diagnosed by machine learning methods

Candidate species	Random Forest (MDA)^*a*^		EdgeR (P)^*b*^	
	Blood^*c*^	CSF^*d*^	Blood^*c*^	CSF^*d*^
Escherichia coli	3.03	0.004	2.93E−04	0.11991
Klebsiella pneumoniae	0.43	0.002	2.98E−03	4.46 E−04
Thermothelomyces thermophila	3.07	<0.002	3.82E−04	1.63E−04
Lactobacillus acidophilus	<0.43	<0.002	NA	1.04E−08
Staphylococcus haemolyticus	2.29	<0.002	0.02	5.60E−05
Lactococcus lactis	<0.43	<0.002	NA	1.26E−07

a MDA, mean decrease accuracy value.

b P, *P* value. NA, not available.

c Blood, comparing NB and PB.

d CSF, comparing IN and PC.

On the basis of the above two screening assays, we screened representative microbiota related to meningitis, which was regarded as a signature in patients. There were significant differences in the relative abundances of Escherichia coli, Klebsiella pneumoniae, Thermothelomyces thermophila, Lactobacillus acidophilus, Staphylococcus haemolyticus, and Lactococcus lactis (Table 1) in patients compared to those in the healthy and undiagnosed control subjects. Our screening criteria were a *P* value less than 0.01 and MDA ranked in the top 10. These results showed that the representative microbiota constituents in the blood and CSF can serve as biomarkers to identify meningitis patients.

### The representative microbiota can serve as a specific signature for clinical stratification.

**FIG 3:**
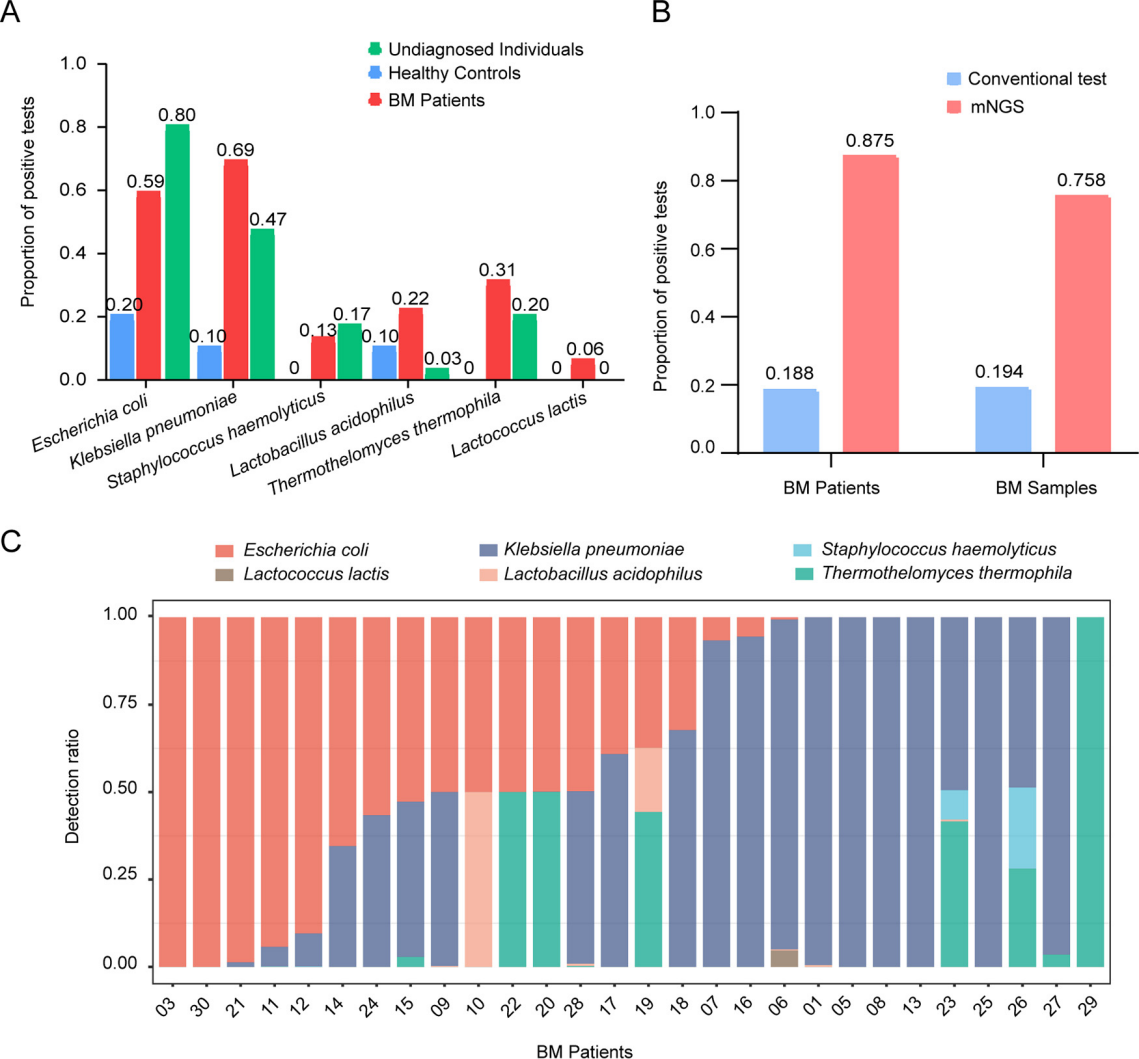
The candidate microbiota presented specific patterns and can serve as a signature for clinical stratification. (A) Detection rate of the candidate microbiota in different groups: healthy controls, bacterial meningitis (BM) patients, and undiagnosed individuals. (B) Bar plot showing the positive ratio of the candidate bacteria in two different groups: patients and samples. (C) Bar plot denoting the relative abundance of six bacteria at the species level for each sample. The *x* axis indicates the number of patients with bacterial meningitis. The *y* axis represents the detection ratio of target microbiomes based on the average relative abundance.

We wondered whether the candidate microbiota distribution was discrepant among different populations. Therefore, we first monitored the detection rate of the candidate microbiota in different groups based on the sum of the nonredundant frequencies of each species. The detection rates of Escherichia coli and Klebsiella pneumoniae in infected individuals were higher than those in healthy children, and Staphylococcus haemolyticus, Thermothelomyces thermophila, and Lactococcus lactis appeared only in infected individuals (Fig. 3A). In addition, we also tested the positive ratio of the candidate microbiome at two different levels: patient and sample size. The positive rate of mNGS was obviously higher than that by conventional detection. For the patient cohort, the detection rate using mNGS was 0.875 (*n* = 28/32) (Fig. 3B, left, red), and the detection rate by the conventional approach was 0.188 (*n* = 6/32) (Fig. 3B, left, blue). For the specimen cohort, the positive detection rate using mNGS was 0.758 (*n* = 47/62) (Fig. 3B, right, red), and the positive detection rate by the conventional approach was 0.194 (*n* = 12/62) (Fig. 3B, right, blue). Objectively, compared with traditional techniques for cultivating live organisms, the mNGS method can detect all remnants of DNA, which may make its detection sensitivity appear to be greater. These findings suggested that mNGS is effective and that the representative microbiota might be regarded as a reliable marker.

To further explore the distribution of the target microbiome in BM children, we measured the relative abundance of a single bacterium (species counts/microbiota counts) in each patient. Interestingly, we found a specific combination pattern of the candidate microbiome. The number and species of bacteria among meningitis cases were obviously diverse (Fig. 3C, BM patients 03, 10, 22, 08, and 29), but they showed a certain trend that some patients had Escherichia coli as the dominant species, while others had Klebsiella pneumoniae as the dominant species (Fig. 3C, Escherichia coli dominates patients 03, 30, 21, and 11 while Klebsiella pneumoniae dominates in patients 01, 05, 08 and 13). These results strongly indicated that the representative microbiota could serve as a signature for clinical stratification.

### The representative microbiota can reflect the inflammatory status and infection degree.

**FIG 4:**
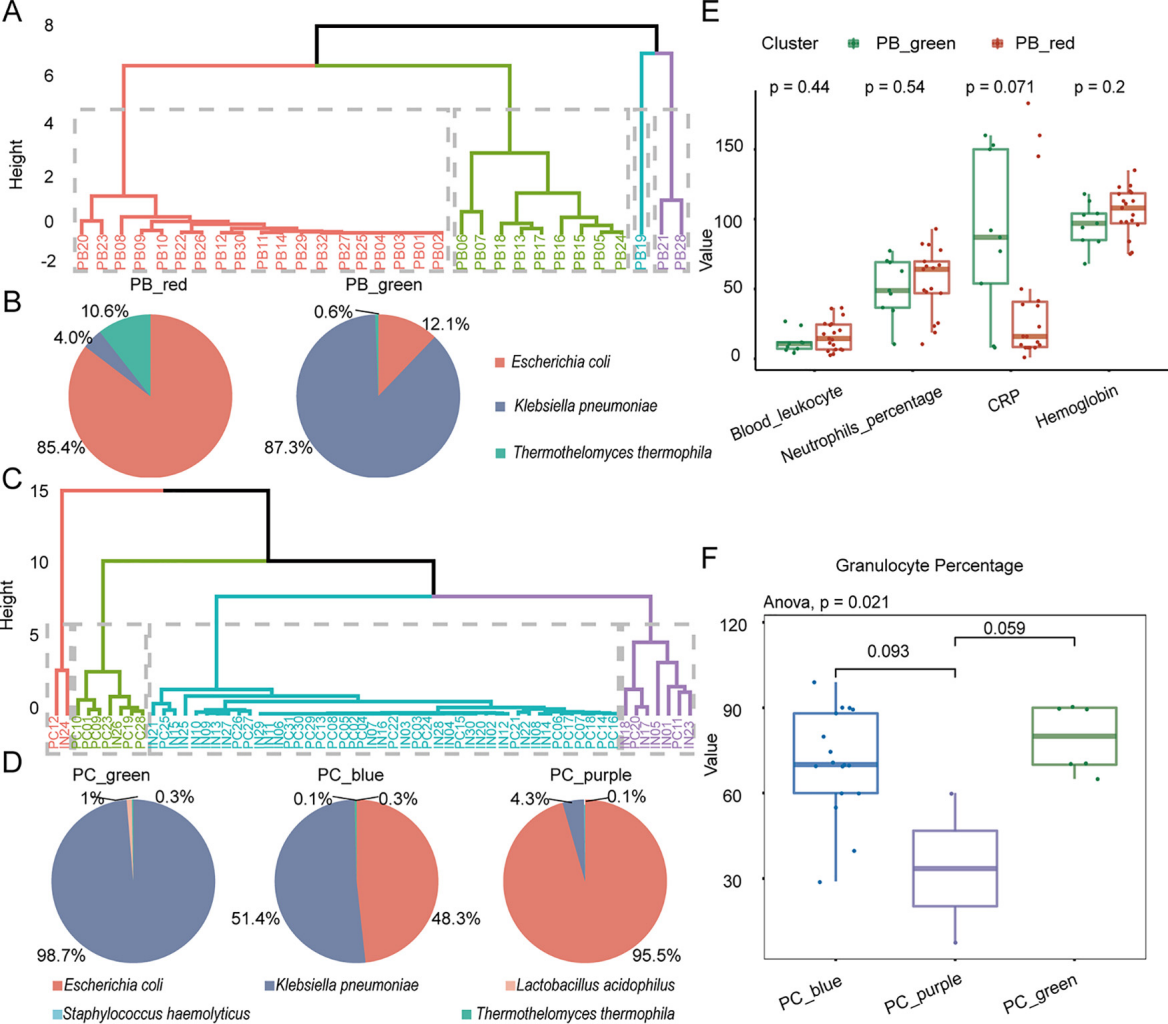
The candidate microbiota can reflect the inflammatory status and infection degree. (A) Four subsets (PB_red, PB_green, PB_blue, and PB_purple) clustered by the k-means method based on candidate microbiota in patient blood. (B) Pie chart showing the dynamic distribution pattern of candidate microbiota for each subgroup in panel A. (C) Four subsets (PC_red, PC_green, PC_blue, and PC_purple) clustered by the k-means method based on candidate microbiota in patient CSF. (D) Pie chart showing the dynamic distribution pattern of the target microbiome. (E) PB_green, with a predominance of Klebsiella pneumoniae, had higher CRP in blood than PB_red. (F) The proportion of granulocytes was significantly different in PC_blue, PC_green, and PC_purple (*P* = 0.021, annova.test), and patients with a predominance of Klebsiella pneumoniae (PC_green and PC_blue, panel D, left and middle) had a higher proportion of granulocytes in their CSF than those (PC_purple, panel D, right) with a predominance of Escherichia coli. Note that the Wilcoxon test was used for comparison between the two groups.

On the basis of the above patterns, we attempted to distinguish patient subgroups. Fortunately, we identified the optimal representative microbiota for different patient subsets. Patients were divided into four distinct clusters based on the combination of Escherichia coli, Klebsiella pneumoniae, and *Thermothelomyces thermophila* in blood (Fig. 4A). The data showed that the bacterial characteristics in PB_red (Fig. 4B, left) and PB_green (Fig. 4B, right) were significantly different, and the dominant pathogens were E. coli and K. pneumoniae, respectively.

Similarly, we also found that Escherichia coli, Klebsiella pneumoniae, *Thermothelomyces thermophila*, Staphylococcus haemolyticus, and Lactobacillus acidophilus in CSF were able to classify patients into four distinct subgroups (Fig. 4C). Additionally, the CSF of undiagnosed patients (IN) served as an input in k-means clustering for referring to meningitis patients in the same subgroups. The data showed that the bacterial composition pattern in PC_green (Fig. 4D, left), PC_blue (Fig. 4D, middle), and PC_purple (Fig. 4D, right) were obviously different, with a predominance of K. pneumoniae, K. pneumoniae plus E. coli, and E. coli, respectively. We asked whether the different microbial compositions were associated with the clinical status of patients.

To confirm the relationship between the target microbiome and the patient’s infection status, we analyzed the potential correlation between its distribution pattern and clinical testing indexes in blood and CSF (Table 2), respectively. The results showed that patients with a predominant abundance of Klebsiella pneumoniae (PB_green, Fig. 4B, right) had higher blood CRP levels (Fig. 4E, CRP) than patients (PB_red, Fig. 4B, left) predominated by Escherichia coli (Fig. 4E). A recent study described that CRP, a vital inflammatory mediator, has high specificity in the CSF of children with BM (18). Consequently, the results suggested that the target microbiota with different composition patterns can reflect the inflammatory degree of patients. A higher abundance of K. pneumoniae indicates an increase in the degree of inflammation in BM patients.

**TABLE 2:** Clinicopathological characteristics of bacterial meningitis patients

Specimen and clinical test indicator	PB_red	PB_green	PC_blue	PC_green	PC_purple
Blood					
Leukocytes	21.58 ± 21.04	5.72 ± 2.2			
Neutrophils (%)	81.7 ± 41.15	47.75 ± 1.63			
CRP	27.55 ± 16.19	115 ± 53.74			
Hemoglobin (g/liter)	98 ± 0	93.5 ± 13.44			
CSF					
Leukocytes (× 10^6^/liter)^*a*^			664 ± 927.72	1,802.5 ± 1,665.24	1,733.87 ± 97.06
Granulocytes (%)			70 ± 21.21	70 ± 12.01	41 ± 41.01
Monocytes (%)			30 ± 21.21	30 ± 12.04	21.02 ± 12.7
Protein (mg/liter)			2,223 ± 268.7	840 ± 622.25	731.13 ± 153.97
Glucose (mmol/liter)			1.23 ± 0.74	3.19 ± 1.27	2.23 ± 1.35
HTS_score^*b*^			5 ± 1.41	2.5 ± 2.12	NA^*c*^

a Note that the CSF leukocyte unit of measurement is the value in the table × 10^6^.

b The HTS score is a scoring system that comprehensively evaluates patients based on factors such as coma, hypothermia, convulsions, shock, age, glucose amount, and visit time. The higher the score, the more serious the patient's disease state.

c NA, not available.

The proportion of granulocytes was significantly different in three subgroups (Fig. 4F, *P* = 0.021, annova.test), and patients with Klebsiella pneumoniae as the dominant pathogen (PC_green and PC_blue, Fig. 4D, left and middle) had a higher proportion of granulocytes in their CSF than those (PC_purple, Fig. 4D, right) with Escherichia coli as the dominant species. Studies have proposed that a predominance of neutrophil granulocytes suggests BM. However, in viral and chronic infections, lymphocytes and monocytes prevail (19). Granulocytes include neutrophils, eosinophils, and basophils, and the increased proportion of granulocytes indicates that bacterial infection tends to be more serious. Therefore, the predominance of K. pneumoniae suggests a severe infection. These results suggest that the target microbiota can be used as an indicator of infection and inflammation in BM patients.

### The blood has the same detection power as cerebrospinal fluid in patients with a predominance of Klebsiella pneumoniae.

**FIG 5:**
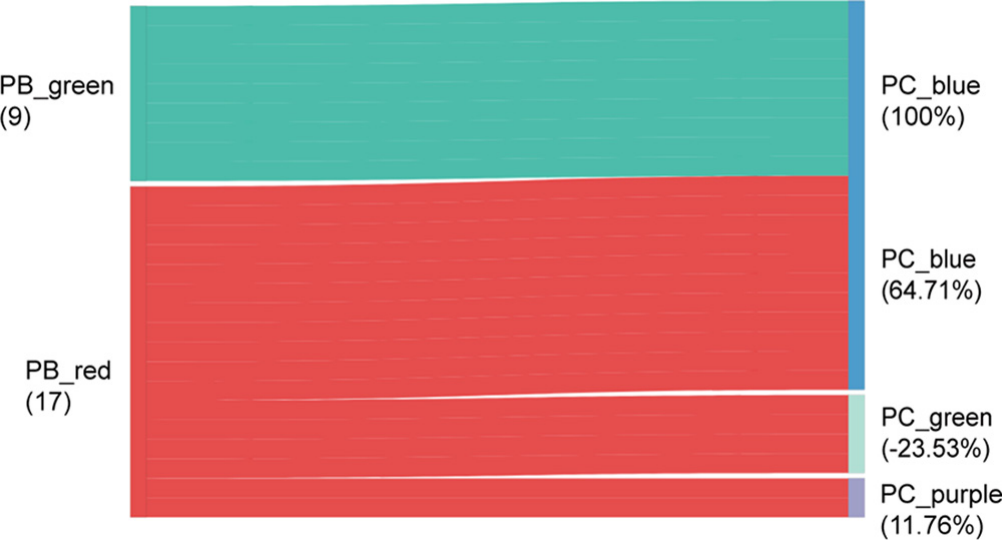
Blood of patients with a predominance of Klebsiella pneumoniae have the same detection power as their cerebrospinal fluid. A sankey diagram showing the flows of patient subgroups. All patients in PB_green (*n* = 9, 100%) were present in PC_blue (*n* = 22), which was dominated by K. pneumoniae. Most children with meningitis with a predominance of Escherichia coli (*n* = 13) in PB_red (*n* = 17, 76.47%) were assigned to the PC_blue (*n* = 22) and PC_purple subgroups (*n* = 2). Four cases in PB_red (*n* = 17, −23.53%) were assigned to PC_green, whose primary species was K. pneumoniae.

Previous results have demonstrated that the dynamic distribution of target microbiota in specimens can reflect the infection and inflammation status of BM patients. We wondered whether blood has the same test effect as CSF; that is, can blood replace CSF in pathogen detection? Then, we analyzed the distribution consistency of the candidate microbiota in different patient subpopulations, taking blood subsets (PB_green and PB_red, Fig. 5, left) as references. All patients in PB_green (*n* = 9, 100%) were assigned to PC_blue (*n* = 20), which was dominated by K. pneumoniae. Most patients with a predominance of E. coli (*n* = 13) in PB_red (*n* = 17, 76.47%) were assigned to the PC_blue (*n* = 20) and PC_purple subgroups (*n* = 2); however, four cases in PB_red (*n* = 17, −23.53%) were assigned to PC_green, whose primary species was K. pneumoniae. In short, the consistency of blood and CSF in patients (PB_green) with a predominance of K. pneumoniae was 100%, while the consistency was only 52.94% in the PB_red group. Consequently, these data show that the blood specimens of patients with a predominance of K. pneumoniae has the same detection power as their CSF specimens.

### The meningitis-related microbiota from the blood and CSF is associated with metabolic pathways.

**FIG 6:**
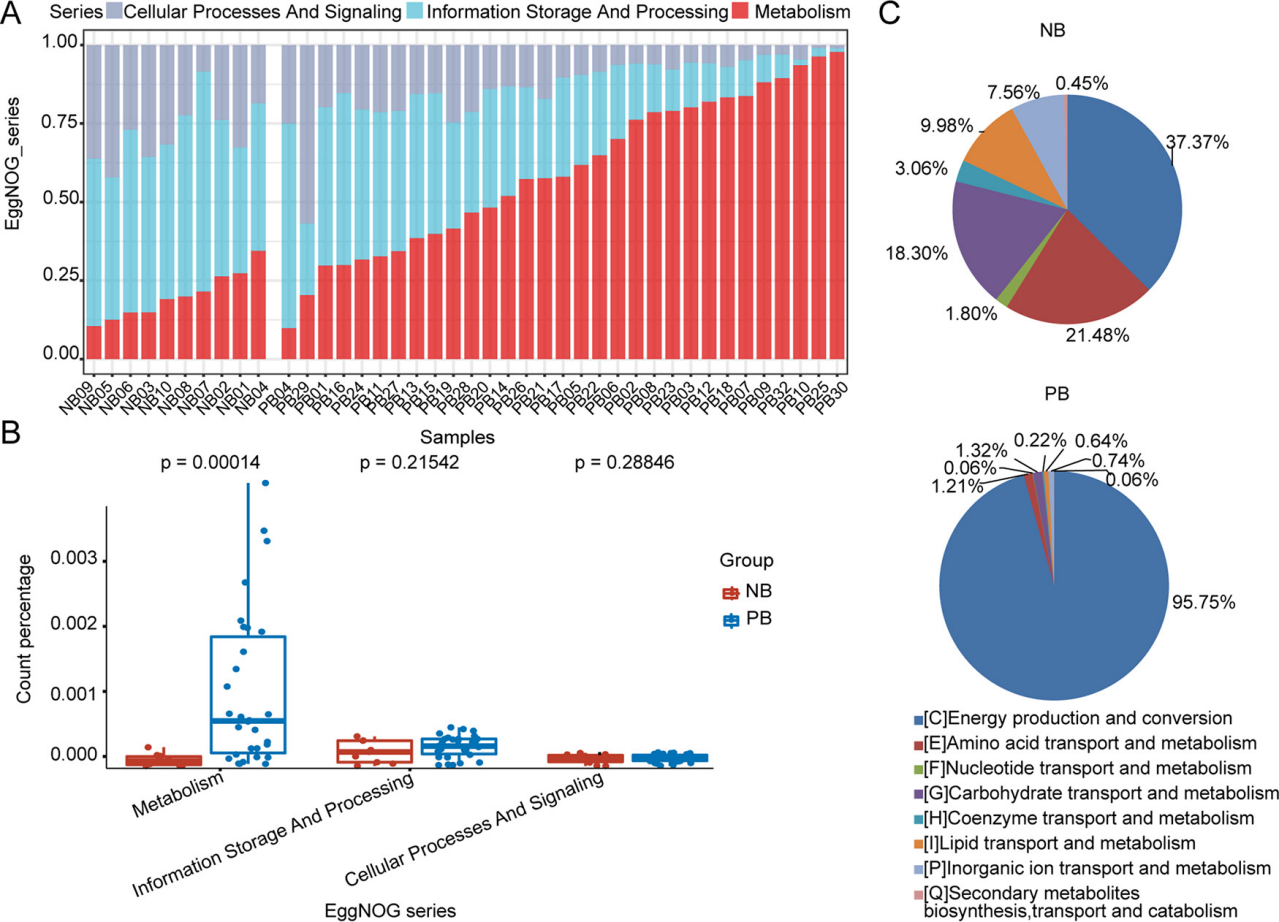
Functional characterization of the candidate microbiome between meningitis patients and healthy controls. (A) Cumulative relative abundance of metabolic functions enriched in the blood microbiota of children with BM. (B) Boxplot showing metabolic functions enriched in PB (*P* < 0.05, Wilcoxon rank sum test). The horizontal bars within boxes represent medians. The tops and bottoms of the boxes represent the 75th and 25th percentiles, respectively. The upper and lower whiskers extend to data no more than 1.5× the interquartile range from the upper edge and lower edge of the box, respectively. The numbers of replicated samples in this figure are as follows: *n* = 10 for NB and *n* = 31 for PB. (C) Pie charts showing proportions of different terms on metabolism functional categories in the NB and PB groups. The sector represents the proportion of each kind of pathway, and the percentages were calculated based on the mean relative abundance of the microbiomes.

It has been demonstrated that microbial imbalances can induce systemic metabolic alterations (20, 21). Carbohydrate and amino acid metabolism functions are overrepresented in meningitis (22, 23). Recent studies have described that reactive oxygen species (ROS), reactive nitrogen species (RNS), and peroxynitrite are produced in large amounts during pneumococcal meningitis, activating cellular energy depletion, which in turn causes massive meningeal inflammation (24). On the basis of previous studies, we hypothesized that meningitis-causing bacteria have the ability to shape metabolic pathways and influence inflammatory conditions (25). We compared the proportion of microbial reads assigned into three main functional categories: cellular processes and signaling, information storage, and processing and metabolism. A clear distinction in microbial function, especially in metabolism, was observed between patients and controls (Fig. 6B).

Notably, most PB-enriched microorganisms were especially enriched for metabolism (Fig. 6B, Wilcoxon rank sum test, *P* = 0.00014), particularly for energy production and conversion. Other functional categories, including amino acid transport and metabolism, nucleotide transport and metabolism, carbohydrate transport and metabolism, coenzyme transport and metabolism, lipid transport and metabolism, inorganic ion transport and metabolism, and secondary metabolites, were depleted in the PB group (Fig. 6C). These findings reflected the alteration of microbial metabolic activity in patients. Studies revealed that neonatal antibiotic treatment led to imbalance of gut and skin microbiota, which increased murine susceptibility to experimental psoriasis (26). Antibiotic exposure in infancy was found to be a meaningful factor associated with microbial dysbiosis in the guts of babies (27). It was reported that bacterial abundance decreased by one third in the gut of healthy people treated with ciprofloxacin, which included decreasing taxonomic richness, reduced diversity, and falling evenness of the community (28). In addition, ciprofloxacin also affects the composition of the host’s gut microbiota (29). These changes in the microbiomes might influence host physiology and status because the close connection between the microbiota and the host (30). Ilseung Cho and colleagues found that administration of antibiotic intervention improved metabolic hormone levels in young mice (31). Copies of key genes were observed; these genes were involved in the alterations of short-chain fatty acid production, colonic short-chain fatty acid levels, and the regulation of hepatic metabolism of lipids and cholesterol. One study reported that antibiotic intervention disrupted the microbiota, and further altered the host metabolism and adiposity (32).

## DISCUSSION

This study extends the knowledge of the blood and CSF microbiome in children with BM. Our research represents the first report to explore the influence of the meningitis microbiota on clinical manifestations. We performed a comprehensive analysis of the BM microbiome in blood and CSF. Due to antibiotic treatment, much lower microbial diversity was observed in meningitis patients than in untreated patients. Nevertheless, we identified several important species that may play critical roles in meningitis. Furthermore, the blood and CSF cohorts had a similar microbiome signature, with a specific microbiota that was reported individually as meningitis pathogens (18, 33–42). Overall, we detected candidate microbiota that can represent the characteristics of the patient population and divided patients into distinct subgroups. We recognized Escherichia coli, Klebsiella pneumoniae, and Thermothelomyces thermophila as specific signatures in blood and identified Escherichia coli, Klebsiella pneumoniae, Thermothelomyces thermophila, Staphylococcus haemolyticus, and Lactobacillus acidophilus in CSF to be of predictive value for clinical stratification. The target microorganisms showed dynamic and regular changes among different subgroups, and the consistency was highest in the blood and CSF of patients with a predominance of Klebsiella pneumoniae. Remarkably, we expounded for the first time that the microbiota composite pattern was associated with CRP in blood and the granulocyte proportion, supporting a valuable role of the candidate microbiome in reflecting meningitis infection and the inflammatory state.

Escherichia coli is the most common Gram-negative bacillus that causes neonatal meningitis (35). Hematogenous spread is the cause of most cases of E. coli meningitis (43, 44). Considering the plethora of E. coli serotypes, it is striking that E. coli strains with K1 capsular polysaccharides are mainly (about 80%) isolated from neonatal E. coli meningitis (45–47). Studies demonstrate that meningitis-causing E. coli invades human brain microvascular endothelial cells (HBMECs) and transmigrate through HBMECs via an enclosed vacuole (48). Klebsiella pneumoniae is an opportunistic pathogen, which mainly affects people with weakened immune systems and is prone to causing nosocomial infections (38, 49). The abuse of broad-spectrum β-lactamase or carbapenemase leads to the multidrug resistance phenotype of K. pneumoniae, which makes appropriate antibiotic treatment difficult (50, 51). An observational study reported that of 1,859 children with meningitis, 9 cases (0.48%) of K. pneumoniae meningitis were registered in the French national registry (37). Although five cases of Thermothelomyces thermophila infections have been reported in France (34), there is no report of *Thermothelomyces thermophila* in BM children. We first monitored *Thermothelomyces thermophila* DNA in bacterial meningitis patients, although it did not occupy an absolute advantage. Researchers reported a clinical case of drain-associated meningitis caused by methicillin- and linezolid-resistant Staphylococcus haemolyticus (40). In patients with neonatal meningitis, S. haemolyticus has a reduced susceptibility to vancomycin (18). In our study, S. haemolyticus appeared in three BM children treated with ceftriaxone sodium and meropenem. This phenomenon suggests that S. haemolyticus may be less sensitive to other antibiotics, and researchers need to further explore and confirm its resistance mechanism. Lactobacillus acidophilus caused bacteremia in people with weakened immunity such as elderly diabetic patients (42). In summary, not only are the target microorganisms likely or determined to be the pathogenic microorganisms of bacterial meningitis, but their composition may also be used to monitor the effect of antibiotic intervention in patients with bacterial meningitis or provide a reference for clinical diagnosis.

Studies revealed that neonatal antibiotic treatment leads to imbalance of gut and skin microbiomes, which increased murine susceptibility to experimental psoriasis (26). Antibiotic exposure in infancy was found to be a meaningful factor associated with microbial dysbiosis in the guts of babies (27). It was reported that bacterial abundance decreased by one third in the gut of healthy people treated with ciprofloxacin, which included the decreasing taxonomic richness, reduced diversity, and falling evenness of the community (28). In addition, ciprofloxacin also affects the composition of the host’s gut microbiota (29). A study reported that antibiotic intervention disrupted the microbiota, and further altered the host metabolism and adiposity (32). Besides influences on metabolism, the microbiomes also interacted with the host immune system. In other words, disturbances of microbiome might affect the development of inflammatory diseases potentially (31). Researchers confirmed that penicillin caused changes in metabolism and affected the expression of immune-related genes (32). The above studies are consistent with our conclusion that the composition of microorganisms is not only closely related to the host metabolism but also reflects the host inflammation.

The most reliable way to diagnose meningitis is to obtain a cerebrospinal fluid (CSF) specimen by lumbar puncture (LP) for analysis (52). However, some patients such as preterm infants are not suitable for lumbar puncture because of the risk of spinal hematoma and herniation (53–55). If blood can replace cerebrospinal fluid as the main carrier of disease diagnosis and treatment monitoring for some patients, even a minority of patients, our studies could contribute to reducing the risk of further clinical deterioration.

Taking into account the suggestive nature of the results related to the small sample size, we compared the distribution of target microbiomes in all patient subgroups. In the PB_purple, PB_red, PB_blue, and PB_green patient groups (see Fig. S1A in the supplemental material), the proportion of Escherichia coli gradually decreased, while the proportions of Klebsiella pneumoniae and *Thermothelomyces thermophila* increased, but not continuously. In the PC_purple, PC_red, PC_blue, and PC_green patient groups (Fig. S1B), the proportion of E. coli gradually decreased, which was consistent with the results of blood analysis. The proportion of K. pneumoniae was increasing gradually, but continuously. In short, considering the small sample size of the patient group, the analysis results could better show the dynamic changes of microbial distribution, especially the results based on cerebrospinal fluid showed better regularity and continuity.

Due to the rapid onset of BM, the vast majority of children are initially treated with antibiotics. It has been reported that approximately 35% of children with BM are treated with antibiotics before lumbar puncture because a delay in antibiotics can increase the rate of mortality (56). Treatment of children with antibiotics for more than 12 h showed a significant reduction in the positive rate of conventional CSF culture versus antibiotic use for less than 4 h (29). Our study showed that nucleic acid sequences of pathogenic bacteria in BM children with antibiotic intervention could still be detected by mNGS even when the traditional method is limited. As shown in the results, the detection efficiency of mNGS was 4.66 and 3.91 times higher than that of traditional methods in terms of patients and sample size, respectively (Fig. 4B). This result suggests that metagenomics can be used as a complementary approach to conventional detection.

According to Chinese neonatal BM treatment experience, meropenem (40 mg/kg of body weight) is generally used for Klebsiella pneumoniae, with an interval of 8 h and a course of treatment lasting at least 21 days. However, the mean time of meropenem treatment in patients (patients 5, 6, 7, 13, 15, 16, 17, and 24; Fig. 4B, PB_green, 8/9) was only 3.46 days. Notably, child 18 (Fig. 4B, PB_green, 1/9) was given meropenem for 33 days, with 0.2 g each time. K. pneumoniae in his blood (counts = 652) accounted for only 35.47% of the candidate microbiota versus 64.53% for Escherichia coli, while in CSF, K. pneumoniae (counts = 1,532) accounted for 100%. Additionally, the recommended treatment for E. coli is ampicillin in combination with a broad-spectrum cephalosporin (cefotaxime or ceftazidime). These results indicate that the pertinence of empirical antibiotics needs to be improved, and the treatment strategy should be adjusted in a timely manner according to the dynamic changes in the microbiota. In addition, the incongruent response of blood and CSF to antibiotics may be attributed to the blocking effect of the BBB and the BCSFB. Therefore, the most appropriate drug regimen for patients with BM remains to be further discussed.

Clinical manifestations of BM are nonspecific and may include breast rejection or vomiting, abnormal body temperature, irritability, lethargy, low muscle tone, and even seizures. Due to the lack of representative clinical indicators, it is difficult to achieve accurate diagnosis. Subsequently, the existing metagenomics studies based on meningitis case diagnosis (6, 7, 11–13) have laid a foundation for thoroughly investigating the differential pathogenic bacteria in the patient population. We classified patients into subgroups based on their specific microbiota pattern in blood and CSF. We found that Klebsiella pneumoniae had a consistent distribution pattern in blood and CSF; that is, patients with a high abundance of K. pneumoniae in blood would also have a high abundance of K. pneumoniae detected in CSF. In addition, the target microbiome affected the level of CRP in blood and the proportion of granulocytes (monocytes) in CSF, which further influenced the degree of infection and inflammation. Studies have shown that bacteria invade the body of children and enter intracranially through the BBB (cross-cellular pathway, paracellular pathway, etc.). The proliferation of pathogenic bacteria activates the host immune response, leading to the release of a large number of inflammatory factors, ultimately causing severe brain damage (15, 57).

Overall, our research indicates a new direction for improving the accuracy of clinical drug intervention and provides an important reference for disease risk and prognosis assessment of bacterial meningitis.

## MATERIALS AND METHODS

### Subjects and sampling.

Subjects aged 0 to 14 years were admitted from the Department of Infection, Beijing Children’s Hospital, from August 2014 to February 2017. This research compared 31 children with bacterial meningitis (BM) to 10 healthy children based on blood data and compared 31 children with meningitis to 30 undiagnosed individuals based on CSF data. The criteria for eligibility in this study follow: (i) a diagnosis of bacterial meningitis, (ii) age from 0 to 14 years old, (iii) treatment with antibiotic(s), (iv) a discharged diagnosis of improvement, (v) collection of at least 350 μl blood, and (vi) collection of at least 350 μl CSF. All conventional detection of CSF was completed by the Laboratory Department of Beijing Children’s Hospital. Six patients had a positive pathogenic test result before sample collection, and 81.25% patients had a negative result. Thirty patients with undiagnosed infections were treated as positive controls, and 10 healthy children were treated as negative controls. The CSF and plasma samples of the first lumbar puncture of all meningitis patients were tested by metagenomic next-generation sequencing (mNGS) and traditional tests. All bacterial meningitis patients have received antibiotic intervention, and each child received an average of four antibiotics and 15 days of treatment. Among all antibiotics, cephalosporins, meropenem, vancomycin, amoxicillin, and fusidic acid were administered more frequently. Among them, cephalosporins mainly include cefaclor, cefdinir, cefepime, cefmenoxime, and cefoperazone-sulbactam. Due to the relatively high cost, each patient underwent only one mNGS examination.

The study was approved by the Institutional Review Committee of the Shanghai Institute of Nutrition and Health, Chinese Academy of Sciences, and Beijing Children’s Hospital. Written informed consent was obtained from all patients or their legal representatives, which complied with the guidelines of the Shanghai Institute of Nutrition and Health of the Chinese Academy of Sciences and the Beijing Children’s Hospital Institutional Review Committee.

### DNA extraction, library preparation, and untargeted mNGS.

Three hundred fifty microliters of cerebrospinal fluid and plasma were aseptically collected and stored at −80°C. Metagenomic DNA was extracted immediately using Qiagen pathogen lysis tubes and QIAamp UCP pathogen minikit in the National Secondary Biosafety Laboratory (biosafety level 2 [BSL-2]) of the Shanghai Institute of Pasteur of the Chinese Academy of Sciences. Whole-genome amplification was performed using an amplification kit (catalog no. 150345; Qiagen, Germany). All DNA libraries were constructed following the TruSeq protocol (Illumina, San Diego, CA). Whole-genome amplification and library construction were completed within 3 days, and untargeted metagenomic sequencing was performed on the Illumina platform immediately, and yielded 2 × 150-bp paired-end reads. To avoid unexpected sequences, uninfected human CSF samples serving as controls were prepared in parallel, underwent library preparation, and were sequenced in the same run (58). All samples achieved an average coverage of 11.69-fold per base position, and at least 20.57% of the whole-genome regions (hg19) were covered at least 20-fold.

### Metagenomics bioinformatics analysis.

The NGS data set in FASTQ format was processed by an initial preprocessing procedure that consists of low-quality filtering, low-complexity filtering, and adaptor trimming by Trimmomatic (59). Then, unmapped reads were obtained after human host subtraction using samtools and output in bam format (60). The remaining unmapped reads were directly aligned to microbial reference genomes, including viral, archaeal, bacterial, and fungal reference genomes, through Kraken2 which completed taxonomic classification (61, 62). Subsequently, the assessment of species abundance relied on Bracken (Bayesian reestimation of abundance with Kraken) (63). Finally, reads were mapped to the nonredundant protein sequence database (NR database) on a local server by Diamond software (blastx command) (64). Then, mapping results in m8 file format served as inputs into MEGAN6 (community edition) for EggNOG profiling (14). Relative abundance data were profiled in comparison analysis, and clinical relevance analysis was mainly based on R. α-Diversity was calculated based on Shannon and Simpson indexes. Principal coordinate analysis (PCoA) with unconstrained and weighted Bray-Curtis distances was calculated based on species relative abundances. We examined differences in the candidate microbiota constituents with edgeR (*P* < 0.05). edgeR recommends filtering according to the count per million (CPM) value, and species with a CPM value greater than 1 are retained. Then, the number of read counts was converted into log_2_ CPM (log CPM), and the appropriate observation-level weights were calculated by estimating the mean-variance relationship, which was followed along with linear modeling. We used MEGAN6, a comprehensive toolbox for interactively analyzing microbiome data (65, 66), to perform EggNOG functional annotation. Total unmapped reads in blood samples of healthy children and children with meningitis were used as inputs for EggNOG functional characterization using default parameters in MEGAN6. The annotation results of the candidate microbiota were extracted for downstream cumulative abundance assessment. The microbiota composition pattern in each subgroup was assessed by the average relative abundance of each target bacterium in all patients from that subset (mean counts of each bacterium/sum of mean counts of candidate microbiota).

The correlation between the representative microbiota and metabolism was evaluated by the Wilcoxon rank sum test and visualized by ggplot2. Clustering was dependent on the k-means and hclust methods shown by the fviz_cluster command.

10.1128/mSystems.00049-21.2 FIG S2 Characteristics of microbial composition in patients with bacterial meningitis. (A) Patterns of blood microbial composition in patients with bacterial meningitis. (B) Patterns of microbial composition of cerebrospinal fluid in patients with bacterial meningitis. Download FIG S2, TIF file, 1.2 MB. Copyright © 2021 Liao et al. 2021 Liao et al. https://creativecommons.org/licenses/by/4.0/ This content is distributed under the terms of the Creative Commons Attribution 4.0 International license.

## Supplementary Material

Reviewer comments
