# Supplementary figures and images for: Characterization of the Blood and Cerebrospinal Fluid Microbiome in Children with Bacterial Meningitis and Its Potential Correlation with Inflammation
Source: mSystems. 2021 Jun 8;6(3):e00049-21. doi: 10.1128/mSystems.00049-21 (PMC8269202; doi:10.1128/mSystems.00049-21)

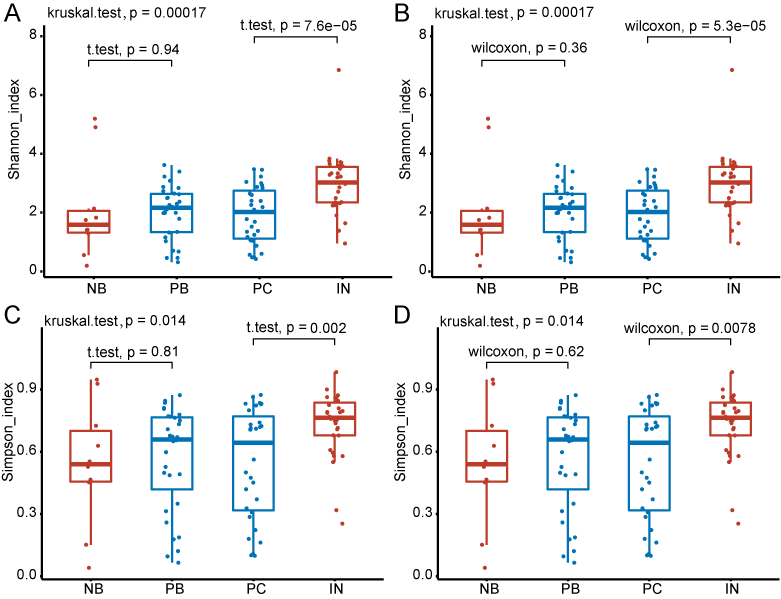

Supplement: FIG S1 [file msystems.00049-21-sf001.tif]

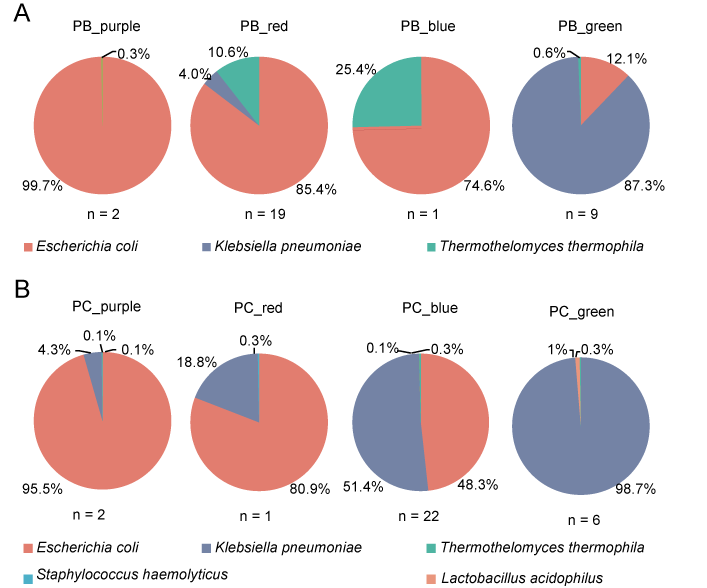

Supplement: FIG S2 [file msystems.00049-21-sf002.tif]
